# Structural Analysis of an Evolved Transketolase Reveals Divergent Binding Modes

**DOI:** 10.1038/srep35716

**Published:** 2016-10-21

**Authors:** Pierre E. Affaticati, Shao-Bo Dai, Panwajee Payongsri, Helen C. Hailes, Kai Tittmann, Paul A. Dalby

**Affiliations:** 1 Department of Biochemical Engineering, Gordon Street, University College London, WC1H 0AH, UK; 2 Albrecht-von-Haller Institute, Göttingen Center for Molecular Biosciences, Georg-August University Göttingen, 37077 Göttingen, Germany; 3 Department of Chemistry, 20 Gordon Street, University College London, WC1H 0AJ, UK

## Abstract

The S385Y/D469T/R520Q variant of *E. coli* transketolase was evolved previously with three successive smart libraries, each guided by different structural, bioinformatical or computational methods. Substrate-walking progressively shifted the target acceptor substrate from phosphorylated aldehydes, towards a non-phosphorylated polar aldehyde, a non-polar aliphatic aldehyde, and finally a non-polar aromatic aldehyde. Kinetic evaluations on three benzaldehyde derivatives, suggested that their active-site binding was differentially sensitive to the S385Y mutation. Docking into mutants generated *in silico* from the wild-type crystal structure was not wholly satisfactory, as errors accumulated with successive mutations, and hampered further smart-library designs. Here we report the crystal structure of the S385Y/D469T/R520Q variant, and molecular docking of three substrates. This now supports our original hypothesis that directed-evolution had generated an evolutionary intermediate with divergent binding modes for the three aromatic aldehydes tested. The new active site contained two binding pockets supporting π-π stacking interactions, sterically separated by the D469T mutation. While 3-formylbenzoic acid (3-FBA) preferred one pocket, and 4-FBA the other, the less well-accepted substrate 3-hydroxybenzaldehyde (3-HBA) was caught in limbo with equal preference for the two pockets. This work highlights the value of obtaining crystal structures of evolved enzyme variants, for continued and reliable use of smart library strategies.

Transketolase (TK) (EC 2.2.1.1) is a ubiquitous thiamine diphosphate (ThDP)-dependent enzyme linking the non-oxidative pentose phosphate pathway and the Calvin cycle^1^. It catalyses the reversible transfer of a two-carbon moiety from a ketol donor to an aldehyde acceptor^2^,^3^. Transketolase has been used to form asymmetric carbon-carbon bonds in biocatalytic organic synthesis^4^,^5^,^6^, where the use of β-hydroxypyruvate (HPA) as the ketol donor renders the donor half-reaction irreversible. The stereoselective synthesis of α,α’-dihydroxyketones can be directed to the synthesis of ketosugars^6^, chiral aminodiols^7^,^8^ and other high-value synthons^9^,^10^,^11^,^12^.

Besides the biocatalytic potential, TK has proven to be a suitable model to evaluate new directed-evolution approaches^13^,^14^,^15^, in which small libraries were found to be effective with several targeting strategies, such that by altering even highly-conserved sites, the substrate specificity of an enzyme could be modified significantly. Using relatively small libraries, 100-fold improvements in efficiency can be achieved compared to error-prone PCR (EP-PCR)^16^ approaches. Such methods have been successfully applied to lipases, for example, to expand substrate-range via combinatorial active-site saturation test (CASTing) or improve thermostability via iterative saturation mutagenesis (ISM)^17^,^18^. Likewise, constrained alphabet approaches have proven successful tools to maximise directed evolution efficiency by creating smaller and/or smarter libraries^14^,^19^,^20^,^21^.

For *E. coli* TK a series of smart library approaches were combined previously in an overall substrate-walking approach, to shift the substrate specificity from phosphorylated to non-phosphorylated polar acceptors^13^, then to non-polar aliphatic substrates^22^,^23^,^24^, and on to hetero-aromatic^25^ and non-polar aromatic substrates^15^,^26^. As shown in Fig. 1, a different library design strategy was used at each stage to guide saturation mutagenesis, including the use of structural and phylogenetic information^13^,^22^, statistical coupling analysis (SCA) methods to target networks for re-stabilisation^14^, and substrate docking^15^. In the latter stage, saturation mutagenesis of two TK active-site residues led to variants, including S385Y/D469T/R520Q, that were active on three benzaldehyde derivatives^15^ (Fig. 2), in contrast to wild-type TK which was active only on non-aromatic aldehydes^25^.

Interestingly, kinetic analysis of S385Y/D469T/R520Q (Table 1) showed that this variant improved the activities towards the three benzaldehyde analogues, 3-formylbenzoic acid (3-FBA), 4-formylbenzoic acid (4-FBA) and 3-hydroxybenzaldehyde (3-HBA), in three different ways^15^. For 3-FBA, the variant displayed a 10-fold improved (lower) *K*_m_, yet only marginally increased *k*_cat_ by 16%, relative to double mutant D469T/R520Q, to give an 11.5-fold increase in *k*_cat_/*K*_m_ at 5400 ± 1490 s^−1^ M^−1^. By contrast, the same variant increased *k*_cat_ towards 4-FBA 8.5-fold, and decreased only *K*_m_ marginally, for a 13.6-fold improved *k*_cat_/*K*_m_. Finally for 3-HBA, the *k*_cat_ and *K*_m_ were both improved from previously unmeasurable values, to give a respectable *k*_cat_/*K*_m_ of 5.4 s^−1^ M^−1^, albeit still with a relatively poor *K*_m_ of 390 mM. These contrasting observations suggested that the positioning and orientation of each of the tested substrates within the active-site was differentially sensitive to the S385Y mutation, and that this was impacting both *K*_m_ and *k*_cat_. Thus evolutionary divergence into distinct binding modes was speculated to have arisen through different orientations of the aldehyde moiety for the three aromatic-aldehyde substrates, relative to the cofactor. Such a mechanism was difficult to confirm by substrate docking into an enzyme structure generated by modelling of three active-site mutations. This also made further rounds of directed evolution more challenging when using structure-docking approaches to guide small library designs, particularly where the different substrates potentially require the design of distinct libraries. To elucidate the impact of the three active-site mutations, we have now determined the crystal structure of S385Y/D469T/R520Q *E. coli* TK. We have then used molecular docking to identify potential substrate-binding mechanisms that led to the divergent kinetic behaviour observed with the benzaldehyde analogues.

## Results and Discussion

### Structural comparison of S385Y/D469T/R520Q and wild-type *E. coli* TK

The crystal structure of S385Y/D469T/R520Q *E. coli* TK in the presence of its cofactors was determined to 1.50 Å resolution with very good statistics (*R*_work_: 12,58%, *R*_free_: 15,69%) (Table 2).

**Figure 3:**
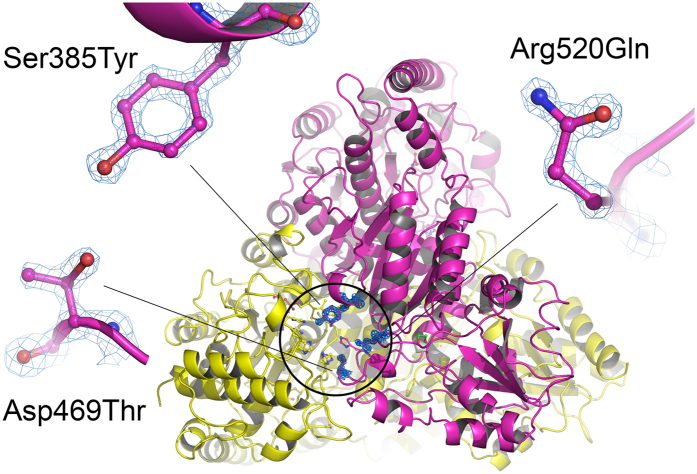
X-ray crystal structure of *Ec*TK S385Y/D469T/R520Q highlighting the mutation sites at the active site (encircled). The homodimer is shown in cartoon representation with individual colors for the two chains (A in yellow, B in magenta). Mutated residues Tyr385, Thr469 and Gln520 are shown in ball-and-stick representation. The calculated electron density of the three amino acid residues is shown at a contour level of 1.5σ in a 2*F*_o_-*F*_c_ map.

The structural analysis of *Ec*TK S385Y/D469T/R520Q could unambiguously confirm the introduced mutations (Fig. 3).

**Figure 4:**
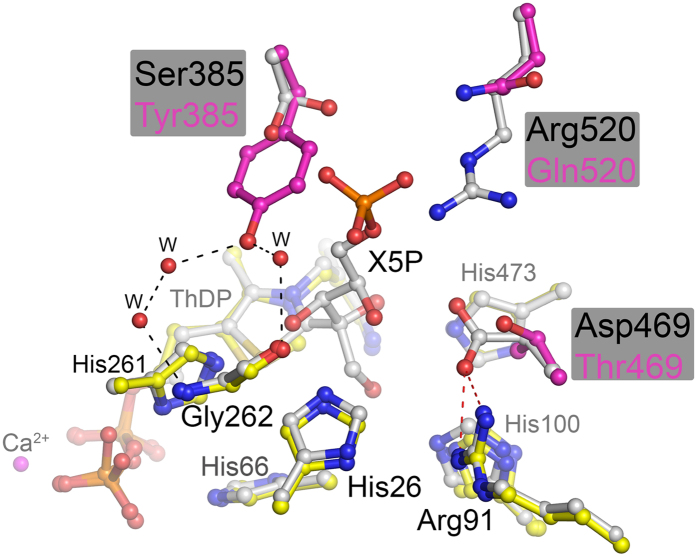
Superposition of the active site of EcTK S385Y/D469T/R520Q (yellow) with that of wild-type EcTK in complex with substrate D-xylulose-5-phosphate (grey). The mutation sites are highlighted in magenta. Note that the salt bridge between Asp469 and Arg91 (red dashes) has been removed in the variant. The OH of introduced Tyr385 H-bonds to residue Gly262 via three water molecules with no known equivalent in the wild-type structure (black dashes).

A structural alignment of the variant with wild-type *E. coli* TK in the resting state (pdb code 1QGD, resolution 1.90 Å)^27^ and in complex with substrate *D*-xylulose-5-phosphate (pdb code 2R8O, 1.47 Å) determined previously^28^ revealed an RMSD (using all 662 residues per chain, and 99% of sequence in PDBeFold^29^) for the dimer of 0.37 Å and 0.40 Å, respectively. This indicates very little structural change resulting from the three mutations. In fact, there was no evidence of any significant shifts in backbone, sidechain or cofactor structure compared to the wild-type protein. Comparison with our previously reported energy-minimised model showed structural differences at all 3 positions, with the aromatic ring of S385Y rotated nearly 90° in the model. Therefore, the observed changes in substrate affinity and catalysis are very likely all directly attributable to the mutational sites themselves. A superposition of *Ec*TK S385Y/D469T/R520Q with *Ec*TK wild-type in covalent complex with substrate *D*-xylulose-5-phosphate (pdb code 2R8O) provides structural insights that can explain the observed functional changes (Fig. 4). The aromatic side chain of introduced residue Y385 is accommodated almost coplanar 7–8 Å above the thiazolium ring of ThDP thus generating a snug π-π stacking binding pocket for aromatic substrates (sandwiched between Y385 and ThDP). The binding of aliphatic phospho-sugar substrates is clearly impeded as the Y385 side chain would clash with the substrate phosphate moiety, and the S385Y mutation also removes a hydrogen-bonding site to the S385 hydroxyl moiety, which is known to interact with phosphate groups in the natural substrates of TK^28^. In the triple variant, the side chain of Y385 is held in place through a network of H-bonding interactions involving 3 water molecules and residue G262. The D469T mutation removed a salt bridge to R91, and replaced it with a hydrogen bond from the T469 hydroxyl moiety. The mutation also created a small pocket in which the R91 guanidinium moiety is more solvent accessible within the enzyme active site, and also available for hydrogen bonding to potential substrates. The phenyl hydroxyl moiety of Y385 instead formed a hydrogen bond to the backbone carbonyl of Gly262. The R520Q mutation created a wider opening to the active-site funnel, which may compensate for the decreased steric access for substrates, introduced by the S385Y mutation.

### Computational docking of aromatic aldehydes into S385Y/D469T/R520Q

**Figure 5:**
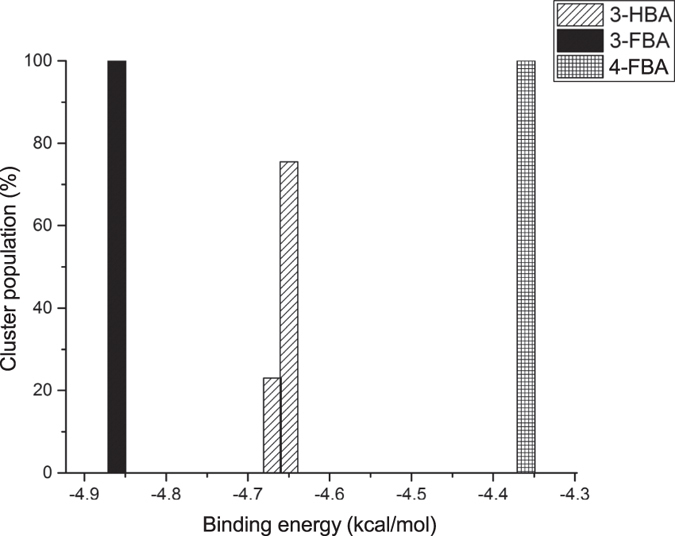
Energy clusters generated by molecular docking of 3-HBA, 3-FBA and 4-FBA into the active site of the triple-mutant transketolase.

Computational molecular docking of polar aromatic substrates 3-FBA, 4-FBA and 3-HBA into the active site of the TK triple-mutant, each produced clusters with very different binding behaviours. After energetic curation, the poses generated for 3-FBA and 4-FBA each produced a single cluster, distinctly different from each other, and with 100% of poses in catalytically productive orientations. Catalytically productive was broadly defined as having aldehyde moieties placed within 4Å of the enamine-ThDP cofactor intermediate. By contrast, 3-HBA resolved into two distinct clusters (Fig. 5), with 77% of poses clustered into a potentially productive orientation (cluster A). The other 23% of 3-HBA poses clustered into a distinctly different binding location (cluster B), with a calculated affinity only slightly higher, but in a non-productive orientation.

**Figure 6:**
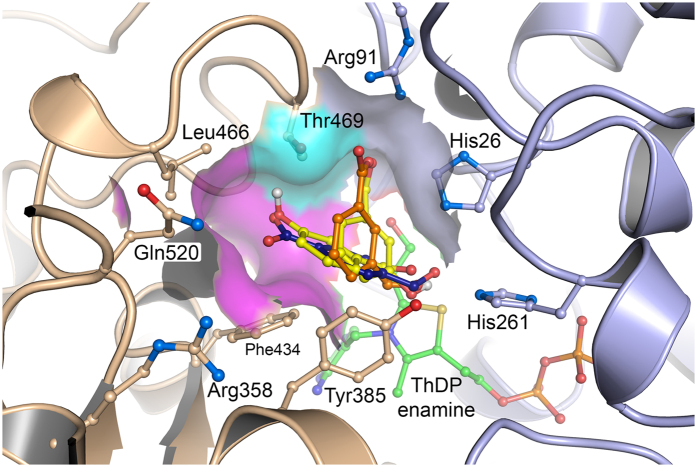
Computational docking of 3-HBA (yellow), 3-FBA (orange) and 4-FBA (blue) into the active site of S385Y/D469T/R520Q *E. coli* transketolase. Oxygen atoms (red) identify the position of the aldehyde moiety relative to ThDP. 3-FBA and the unproductive pose of 3-HBA (cluster B, cyan surface), each hydrogen bond to the R91 and T469 sidechains, while their aromatic rings are positioned at 90° to the F434 ring, and 45° to the Y385 ring. 4-FBA and the productive pose of 3-HBA (cluster A, magenta surface) hydrogen bond to the T469 sidechain, while their aromatic rings are positioned at 45° to the F434 ring, and 90° to the Y385 ring. The two binding pockets are separated by the T469 sidechain.

**Figure 7:**
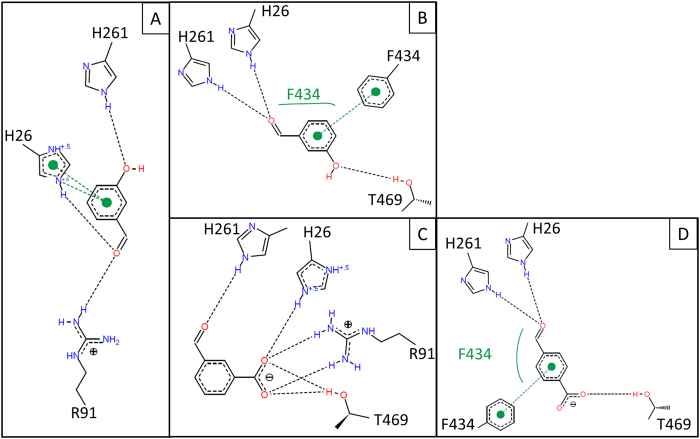
Non-covalent interactions involved in the ligand-TK complex of (**a**) unproductive 3-HBA (**b**) productive 3-HBA (**c**) 3-FBA and (**d**) 4-FBA. Black and green dashed lines respectively refer to hydrogen bond and π-π stacking interactions. Hydrophobic interactions are shown as green solids lines next to the effecting amino acid.

Two distinct active-site binding pockets were observed overall (Fig. 6). The contacts they make with docked substrates are shown schematically in Fig. 7, and summarised in Table 3. Pocket 1 was formed on one face of the T469 sidechain, and docked 3-FBA in a productive orientation, and the less populated 3-HBA cluster B in an unproductive orientation. The aldehyde O-atom of 3-FBA, and the hydroxyl moiety of 3-HBA in cluster B, formed hydrogen bonds to the sidechains of H26 and H216. The carboxylate of 3-FBA, and the aldehyde carbonyl of 3-HBA formed hydrogen bonds to the guanidine moiety of R91, and for 3-FBA only, also to the T469 hydroxyl moiety. The substrate aromatic ring bound edge-to-edge to the F434 ring, in a π-π stack with an interplanar angle between the aromatic ring faces of 90°, and with the F434 ring-edge placed 3.3–3.6 Å below the face of the substrate ring. The Y385 aromatic ring-edge interacted with the substrate ring face, with an interplanar angle of 45°, and the substrate ring-edge positioned 3.7–4.4 Å above the Y385 ring face. This form of π-π stacking at a 45° interplanar angle is suboptimal and lies at an energetic saddle point between the more stable π-π sandwich (0°) and edge-on T-shaped (90°) orientations.

Pocket 2 was formed on the opposite face of T469 to pocket 1, and docked both 4-FBA and the productive pose of 3-HBA, with the aldehyde O-atoms hydrogen bonded to the sidechains of H26 and H216. The hydroxyl moiety of 3-HBA, or the carboxyl moiety of 4-FBA, was oriented towards L466, in close proximity to the T469 β-methyl group, but also with the potential to hydrogen bond to the T469 hydroxyl moiety. The substrate aromatic ring bound edge-to-edge to the Y385 ring, with an interplanar angle of 90°, and with the substrate ring edge placed 3.9 Å below the face of the Y385 ring. The F434 aromatic ring-edge interacted with the substrate ring face, with an interplanar angle of 45°, and the F434 ring edge was positioned 3.3 Å above the substrate ring.

Overall, there was a clear similarity between the binding modes of the two pockets, which docked the substrates with the aldehyde moieties in similar positions, but with an approximately 45° rotation of the ring faces relative to each other, pivoted around the aldehyde moiety position. This 45° rotation resulted from binding of the aromatic substituents into the pockets on either face of T469, where both docking modes provided similar π-π stacking to the F434 and Y385 aromatic rings, with interplanar angles of 90° and 45° respectively for pocket 1, but 45° and 90° respectively for pocket 2. The additional hydrogen bonding to R91 in pocket 1, provided an additional interaction for 3-FBA (and the unproductive 3-HBA mode) that may explain the lower *K*_m_ observed for 3-FBA (Table 1).

The unproductive 3-HBA orientation in pocket 1 described, should not be disregarded entirely because a simple 180° coplanar rotation of the aromatic ring would produce a catalytically productive pose, and also retain a hydrogen bond to R91. This may be a docking artefact based on assigned energy contributions for hydrogen bonding to the carbonyl and hydroxyl moieties of 3-HBA. It could be that either or both orientations of 3-HBA in pocket 1 can populate. Either way, the potential to bind a population of 3-HBA into pocket 1 in an unproductive orientation, in addition to the productive orientations in at least pocket 2, would contribute to the relatively high *K*_m_ observed for 3-HBA. The two clusters may yet resolve into a single binding orientation for 3-HBA if further directed evolution was targeted specifically towards improving activity with 3-HBA.

Inspection of the docked substrates allows us to examine why 3-FBA was only found in pocket 1, and why 4-FBA was only found in pocket 2. 3-FBA cannot bind into pocket 2 with the productive aldehyde-moiety conformation, and the π-π stacking arrangement to F434 and Y385 predicted for 4-FBA as the carboxylate moiety in 3-FBA would be sterically constrained either by H473, or by Y385 for the 180° rotation of the 3-FBA aromatic ring around the bond to the aldehyde moiety. While 3-HBA was predicted to dock in a productive conformation into pocket 2, this was at the expense of a displacement of the aromatic ring edge from the optimal position for π-π stacking to Y385 found for 4-FBA. This observation may also contribute towards the higher *K*_m_ of 3-HBA when compared to 4-FBA.

Conversely, 4-FBA would not favour binding into pocket 1 while retaining the productive aldehyde-moiety conformation, and the π-π stacking arrangement predicted for 3-FBA, as it would not then be able to form the hydrogen bond to R91. Instead, the carboxylate moiety of 4-FBA would be oriented out towards the active-site entrance. Alternatively, to form the hydrogen bond with R91, 4-FBA would not be able to form the same π-π stacking interactions with Y385 and F434 as predicted for 3-FBA.

### Role of mutations in evolution of catalytic activity and specificity

The structural formation of two binding-pockets in the TK triple-mutant, and prediction of differential substrate docking between the two pockets, can be used to rationalise the impact of the combined mutations upon the observed kinetics for the three substrates. We also suggest plausible explanations for the contributions of each mutation *as they occurred* along the evolutionary trajectory from wild type, to D469T, D469T/R520Q, and finally S385Y/D469T/R520Q.

**Figure 8:**
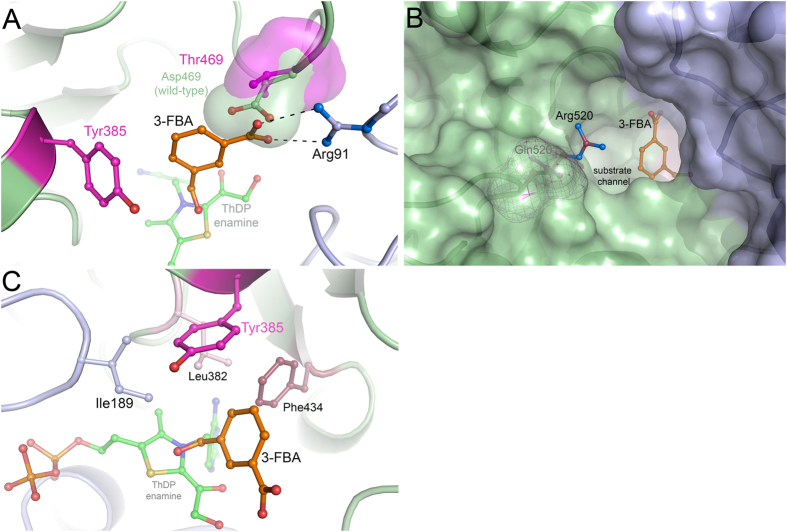
Alignment of the crystal structures of wild-type *E. coli* transketolase, the S385Y/D469T/R520Q triple-mutant, and the pose for 3-FBA (orange) docked into the triple-mutant using Autodock. (**a**) D469 (green) points towards the inside of the active site while D469T (magenta) provides an alternative binding pocket for a phenyl ring. (**b**) The R520 guanidinium group (blue) obstructs substrate access through the binding funnel compared to R520Q (buried in the green surface). (**c**) I189 (purple), L382 (pink), S385Y (magenta) and F434 (red) form a planar hydrophobic pocket which S385Y encloses.

In previous work, the wild-type enzyme was found to be inactive on the three aromatic aldehyde substrates^15^,^26^. The D469T mutation introduced activity towards 3-FBA and to a 10-fold lesser degree towards 4-FBA, which had a 5-fold higher *K*_m_. The acceptance of aromatic substrates induced by the D469T mutation can be explained sterically, as above, by the formation of two binding pockets on either side of T469 in which the γ-hydroxyl and γ-methyl groups of T469 formed a steric barrier between the pockets (Fig. 8a). D469T also created the space for the benzene rings of all three aldehyde substrates, that was otherwise hindered by D469 in the wild type. The lower *K*_m_ for 3-FBA compared to 4-FBA would result from the increased access to R91, which then forms a hydrogen bond to the carboxylate moiety of 3-FBA in pocket 1. The carboxyl moieties of 3-FBA and 4-FBA can each form a hydrogen bond with the γ-hydroxyl group of T469 as this moiety can orient towards either pocket 1 or pocket 2 as necessary.

The R520Q mutation improved the *K*_m_ for both substrates 5–10 fold, yet also decreased *k*_cat_ 2-fold and 25-fold for 3-FBA and 4-FBA respectively. Improvements in *K*_m_ can be explained through improved steric access to the active site (Fig. 8b). However, the mechanism of influence of this mutation on *k*_cat_ is not explained by steric access. Also R520 is 13 Å away from the ThDP cofactor, and hence too far to electronically influence the catalytic residues. However, interaction of R520 with the carboxylate moiety of 3-FBA or 4-FBA would increase the reactivity of the aldehydes by withdrawing electrons, particularly for 4-FBA. Hence the R520Q mutation would be expected to decrease the activity of 4-FBA in particular, as was observed by the decreases in *k*_cat_. However, this effect would also be convoluted with any shift that may have occurred to the binding position and orientation of the aldehydes relative to the cofactor and catalytic residues. If R520 did interact directly with the carboxylate moieties of 3-FBA or 4-FBA in the D469T variant, then this would necessarily pull these substrates further from ThDP and also orient them differently to the docked poses predicted in the triple-mutant. Hence removing this interaction with the R520Q mutation, could also allow more favourable binding close to the ThDP, and in this way contribute to an improved *k*_cat_.

Introduction of the S385Y mutation into D469T/R520Q to produce the triple-mutant creates an enclosed hydrophobic binding-pocket for all three benzaldehyde substrates. All three substrates docked with their aldehyde moieties hydrogen bonded to H26 and H261. As seen in Fig. 8c, this also brings part of their benzene rings into the hydrophobic pocket formed by three coplanar residues I189, L382 and F434. These are members of a previously identified coevolved network of six residues thought to be important for transketolase activity and stability^14^. The Y385 phenyl ring then forms a cap above these residues to enclose the hydrophobic pocket, and the substrate. In Fig. 8c, 3-FBA is shown with the aromatic ring perpendicular to F434. The S385Y mutation, led previously to a 10-fold decrease in *K*_m_ for 3-FBA, without influencing *k*_cat_. This suggests that the binding position and orientation predicted in the docking of 3-FBA to S385Y/D469T/R520Q, was essentially already in place with D469T/R520Q. The improved *K*_m_ thus likely resulted from the new 45° π-π stacking interaction with Y385. By contrast, the *K*_m_ for 4-FBA decreased less than two-fold, but the *k*_cat_ increased almost 10-fold. As above, a strong influence on *k*_cat_ for 4-FBA suggests that the S385Y mutation repositioned this substrate relative to the ThDP-enamine intermediate for more efficient catalysis. 4-FBA formed a favourable 90° π-π stacking arrangement with Y385, which would be expected to improve the *K*_m_ by more than that observed for 3-FBA which only formed the less optimal 45° π-π stacking arrangement. However, the opposite was observed and this is also consistent with an additional movement of the position of the 4-FBA substrate due to the S385Y mutation.

Regardless of the evolutionary route to the kinetics observed in the final triple-mutant S385Y/D469T/R520Q, the end result was a divergence into two binding modes that separate 3-FBA from 4-FBA, but where 3-HBA was still able to bind into both pockets. The structural insights obtained for S385Y/D469T/R520Q and the divergent binding modes, now present different strategies for further engineering of the specificities of each substrate. For example, engineering the specificity towards 4-FBA and 3-HBA, over that for 3-FBA, might best be explored by targeting residues only in binding pocket 2, such as L466. By contrast, re-targeting T469 may allow further fine-tuning of the specificity to all three substrates.

In summary, docking into the new crystal structure for the S385Y/D469T/R520Q variant of TK supports the original hypothesis that directed evolution towards 3-FBA and 4-FBA had led to a divergence in their binding modes. While D469T had already provided a steric boundary between the two binding pockets observed, S385Y introduced π-π stacking interactions differentially for 3-FBA and 4-FBA, and improved the newly evolved activities towards both substrates. The less well accepted substrate 3-HBA, not specifically evolved for, was found to be caught in limbo with the potential for binding to both pockets. Furthermore, this work provides a novel enzyme engineering paradigm whereby a series of semi-rational directed evolution strategies are sequentially built upon and then resolved through crystallography. This will then allow protein engineers to avoid the accumulation of computational errors inherent to models, and provide an informed basis for further semi-rational directed evolution, again guided by molecular docking.

## Methods

All chemical reagents were purchased from Sigma-Aldrich (Aldrich Chemistry, UK) unless otherwise stated.

### Triple-mutant construction

The triple-mutant S385Y/D469T/R520Q was constructed from plasmid pQR791 containing the tktA gene as described previously^13^. Mutations were introduced with mutagenic primers according to the manufacturer’s instructions using the Quickchange kit (Agilent Technologies, USA). Mutations were confirmed by DNA sequencing on both strands.

### Crystallisation and Structure Determination

TK triple variant S385Y/D469T/R520Q was expressed and purified to homogeneity as detailed for the wild-type enzyme^28^. The freshly purified protein was crystallized by the hanging-drop vapor-diffusion method relying on established crystallization conditions^28^ with some minor changes. The triple variant as apo-enzyme was concentrated to 16–20 mg/ml in 50 mM glycyl-glycine buffer, pH 7.9. Afterwards 5 mM ThDP and 5 mM CaCl_2_ were added to the protein solution. 3 μl of protein solution were mixed at a 1 + 1 ratio with reservoir solution containing 17–22% (*w*/*v*) PEG 6000, 2% (*v*/*v*) glycerol, 50 mM glycyl-glycine buffer, pH 7.9. Typically, crystal growth occurred within 4–6 weeks at 8 °C. Before flash-cooling a crystal in liquid nitrogen, it was incubated in a cryoprotectant solution consisting of 20% (*w*/*v*) PEG 6000, 30% (*v*/*v*) ethylene glycol, 10 mM ThDP and 5 mM CaCl_2_ in 50 mM glycyl-glycine, pH 7.9.

A diffraction data set of a single crystal of *Ec*TK S385Y/D469T/R520Q was collected at a wavelength of 1.00 Å at MAX II BEAMLINE I911–3 at Lund (Sweden). Software package Xds was used for data reduction and scaling^30^. The atomic structure of the variant was determined by molecular replacement using the previously determined structure of wild-type *Ec*TK in complex with substrate *D*-xylulose-5-phosphate (pdb code 2R8O) as search model. The crystal structure was built and refined to a resolution of 1.5 Å (*R*_work_: 12,58%, *R*_free_: 15,69%) using programs Coot^31^ and Phenix^32^ respectively. The final refined model and the corresponding structure factor amplitudes have been deposited in the Research Collaboratory for Structural Biology (http://www.rcsb.org) under accession number 5HHT. Figures were prepared using Pymol software.

### Molecular docking

#### Computational protocol

The enamine-ThDP intermediate present in previously docked crystal structures was aligned into the triple-mutant in Wincoot^31^. The transketolase structure was stripped of all crystallographic waters and docked in Autodock 4.2^33^. Ligands were obtained as SMILEs in PubChem and assigned three-dimensional coordinates in PRODRG^34^. The explorable space for docking was defined as a cube 15 Å in length centred at 23.489, 15.398, 5.953 including the entire active-site and omitting surface hydrophobic pockets. For each search, a Lamarckian genetic algorithm was run 200 times with a maximum number of 25 million energy evaluations. Resulting poses were analysed and checked for hydrogen bonding in PyMOL Molecular Graphics System (Schrödinger, USA) and Poseview^35^.

#### Pose curation

Only the most energetically favourable pose-clusters were retained where the energy difference was significant (>0.5 kcal/mol). When a structurally different cluster with similar energy was produced, both were selected. This methodology retained poses that were observed to be potentially “catalytically unproductive”. Catalytically unproductive was taken to mean orientations of the benzene ring that point the reactive aldehyde group away from the enamine-ThDP intermediate.

## Additional Information

**Accession codes:** The final refined model and the corresponding structure factor amplitudes have been deposited in the Research Collaboratory for Structural Biology (www.rcsb.org) under accession number 5HHT.

**How to cite this article**: Affaticati, P. E. *et al*. Structural Analysis of an Evolved Transketolase Reveals Divergent Binding Modes. *Sci. Rep.* **6**, 35716; doi: 10.1038/srep35716 (2016).

## Figures and Tables

**Figure 1:**
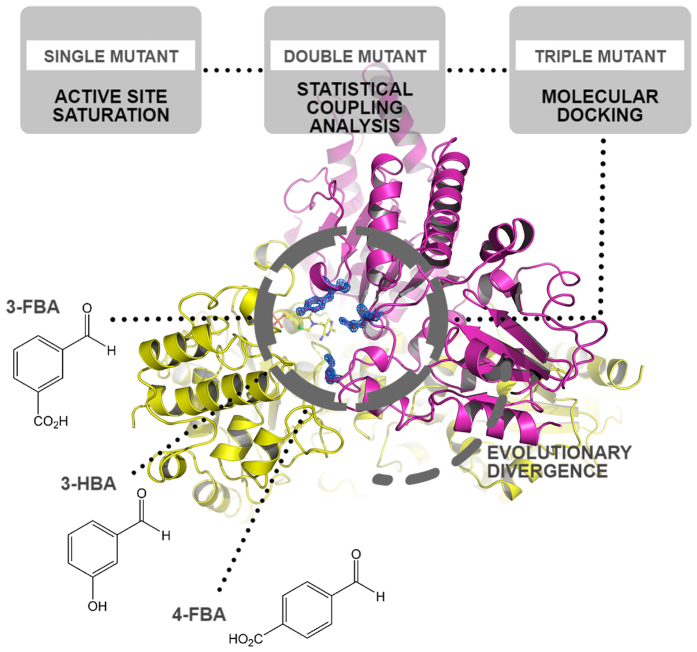
Successive library design strategies used to evolve *E. coli* TK towards aromatic aldehydes.

**Figure 2:**
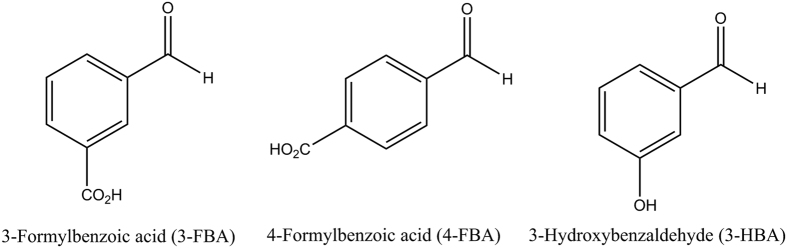
Structures of aromatic aldehydes used with TK variants.

**Table 1:** Kinetic parameters of S385Y/D469T/R520Q towards 3-FBA, 4-FBA and 3-HBA and improvement over the previous variant.

Mutant	Substrate	*K*_M_ (mM)	Fold improvement	*k*_cat_ (s^−1^)	Fold improvement	*k*_cat_/K_M_ (s^−1^M^−1^)	Fold improvement
D469T	3-FBA	56 (10)	N.A.	13.2 (1.5)	N.A.	240 (50)	N.A.
	4-FBA	251 (240)	N.A.	5.0 (4.4)	N.A.	20 (26)	N.A.
	3-HBA	N.A.	N.A.	N.A.	N.A.	N.A.	N.A.
D469T/R520Q	3-FBA	13 (4)	4.3	6.0 (0.75)	0.5	470 (170)	2.0
	4-FBA	25 (8)	10.0	0.20 (0.03)	0.0	8.1 (2.8)	0.4
	3-HBA	N.A.	N.A.	N.A.	N.A.	N.A.	N.A.
S385Y/D469T/R520Q	3-FBA	1.3 (0.4)	10.0	7.0 (0.3)	1.2	5400 (1490)	11.5
	4-FBA	15 (3)	1.7	1.7 (0.1)	8.5	110 (21)	13.6
	3-HBA	390 (10)	N.A.	2.1 (0.2)	N.A.	5.4 (0.1)	N.A.

Standard deviations are shown in brackets. (Data from Payongsri^15^).

**Table 2:** Data collection and refinement statistics.

Data collection	
Wavelength (Å)	1.00
Resolution range (Å)	44.38–1.50 (1.60–1.50)*
Space group	P 21 21 21
Unit cell (a, b, c; α β γ)	89.94 102.04 133.07 90 90 90
Total reflections	797177 (138238)
Unique reflections	190942 (33781)
Multiplicity	4.2 (4.1)
Completeness (%)	97.70 (99.70)
Mean I/sigma (I)	22.7 (7.4)
*R*_merge_ (%)	4.5 (20.1)
CC1/2 (%)	99.9 (94.2)
Wilson B-factor	7.8
Refinement	
*R*_work_ (%)	12,58 (15,09)
*R*_free_ (%)	15,69 (18,99)
Number of non-hydrogen atoms	12312
macromolecules	10364
ligands	198
water	1750
Protein residues	1340
RMS (bonds) (Å)	0.013
RMS (angles) (°)	1.57
Ramachandran favored %)	98.3
Ramachandran allowed (%)	1.7
Ramachandran outliers (%)	0
Clashscore	1.9
Average B-factor (Å^2^)	11.6
macromolecules	8.9
ligands	19.0
solvent	26.4

^*^Statistics for the highest-resolution shell are shown in parentheses.

**Table 3:** Hydrogen bonding (x) and π-π stacking (interplanar angle) profiles for docked poses in the triple-mutant active-site.

	Pocket	H26	H261	R91	T469	Y385	F434
3-FBA	1	x	x	x	x	45°	90°
unproductive 3-HBA	1	x	x	x		45°	90°
4-FBA	2	x	x		x	90°	45°
productive 3-HBA	2	x	x		x	90°	45°
